# Are the minimally invasive techniques the new gold standard in thymus surgery for myasthenia gravis? Experience of a reference single-site in VATS thymectomy

**DOI:** 10.3389/fneur.2024.1309173

**Published:** 2024-02-01

**Authors:** Juan Carlos Trujillo Reyes, Elisabeth Martinez Tellez, Josep Belda Sanchis, Georgina Planas Canovas, Alejandra Libreros Niño, Mauro Guarino, Jorge Hernández Ferrandez, Antonio Moral Duarte

**Affiliations:** ^1^Department of Thoracic Surgery, Hospital de la Santa Creu I Sant Pau, Barcelona, Spain; ^2^Department of Surgery, Faculty of Medicine, Autonomous University of Barcelona, Bellaterra, Spain; ^3^Department of General Surgery, Hospital de la Santa Creu I Sant Pau, Barcelona, Spain

**Keywords:** thoracoscopy, myasthenia gravis, video-assisted thoracic surgery, MG treatment, thymic hyperplasia, thymic tumors

## Abstract

The thymus is the primary lymphoid organ responsible for the maturation and proliferation of T lymphocytes. During the first years of our lives, the activation and inactivation of T lymphocytes occur within the thymus, facilitating the correct maturation of central immunity. Alterations in the positive and negative selection of T lymphocytes have been studied as the possible origins of autoimmune diseases, with Myasthenia Gravis (MG) being the most representative example. Structural alterations in the thymus appear to be involved in the initial autoimmune response observed in MG, leading to the consideration of thymectomy as part of the treatment for the disease. However, the role of thymectomy in MG has been a subject of controversy for many years. Several publications raised doubts about the lack of evidence justifying thymectomy’s role in MG until 2016 when a randomized study comparing thymectomy via sternotomy plus prednisone versus prednisone alone was published in the New England Journal of Medicine (NEJM). The results clearly favored the group of patients who underwent surgery, showing improvements in symptoms, reduced corticosteroid requirements, and fewer recurrences over 3 years of follow-up. In recent years, the emergence of less invasive surgical techniques has made video-assisted or robotic-assisted thoracoscopic (VATS/RATS) thymectomy more common, replacing the traditional sternotomy approach. Despite the increasing use of VATS, it has not been validated as a technique with lower morbidity compared to sternotomy in the treatment of MG. The results of the 2016 trial highlighted the benefits of thymectomy, but all the patients underwent surgery via sternotomy. Our hypothesis is that VATS thymectomy is a technique with lower morbidity, reduced postoperative pain, and shorter postoperative hospital stays than sternotomy. Additionally, VATS offers better clinical improvement in patients with MG. The primary objective of this study is to validate the VATS technique as the preferred approach for thymectomy. Furthermore, we aim to analyze the impact of VATS thymectomy on symptoms and corticosteroid dosage in patients with MG, identifying factors that may predict a better response to surgery.

## 1 Introduction and objectives

The thymus gland, also known as the thymus, is the primary lymphoid organ responsible for the maturation of T lymphocytes, the proliferation of mature T lymphocyte clones, and the control of self-reactive T lymphocytes. During the early years of our lives, within the gland, the activation and, most importantly, the inactivation of T lymphocytes occur, leading to the correct maturation of central immunity (1, 2).

Surgical removal of the thymus, known as thymectomy, is a classic technique first described in 1912 by Ferdinand Sauerbruch, who performed a transcervical thymectomy along with thyroidectomy in a patient with hyperthyroidism and MG. Years later, in 1936, Blalock recommended the exploration of the mediastinum and the performance of a thymectomy in patients with severe MG.

One of the most significant aspects of thymus function is its relationship with autoimmunity. The theories attempting to explain the changes or alterations that cause autoimmune disorders appear too simplistic, especially considering the complexity of the maturation process that takes place in the thymic cortex (3). Currently, there are two scenarios in which thymectomy may be indicated:

- The first scenario is when a thymic tumor is diagnosed. Surgery is the primary treatment, whether or not the tumor is associated with the presence of MG. At the end of 2015, the World Health Organization (WHO) published the 4th edition of the classification of thoracic tumors, where, for the first time, all thymic tumors of malignant etiology are considered (4). This represents a significant advancement in our understanding of thymic tumors and their classification. Fundamental fact when considering treatment, with surgical resection indicated even in advanced stages.
- A second scenario in which thymectomy may be indicated involves selected patients with MG who do not have an associated thymic tumor. The role of thymic surgery in MG has been a subject of debate for many years. A publication by Jaretzki and Sonett in 2008 highlighted the lack of randomized trials supporting the role of thymectomy in patients with MG (5). Another example of this debate occurred in 2010 (6) after the publication of guidelines on the treatment of neuromuscular pathologies, which pointed out the lack of consistent selection criteria and assessments of response after thymectomy.

This controversy surrounding the role of surgery led to a prospective, randomized, multicenter trial, the results of which were published in the NEJM in 2016 (7). This study compared two groups: the first group underwent transsternal thymectomy in addition to prednisone treatment, while the second group received prednisone-only treatment. The results clearly favored the group of patients who underwent surgery, showing improvements in symptoms, reduced need for corticosteroid therapy, and a lower number of recurrences at three years of follow-up.

Following the publication of these results, the role of the thoracic surgeon as part of the multidisciplinary treatment of MG regained importance. The findings from the NEJM publication and the new WHO classification reaffirmed the role of thymectomy both in the treatment of MG patients and in those diagnosed with thymic tumors.

From a surgical perspective, one of the recent advancements has been the progressive and successful implementation of minimally invasive techniques. Initially, VATS and later RATS surgery have become the preferred techniques for most thoracic procedures. However, have we truly validated these techniques in thymus surgery, and what advantages do they offer over the traditional choice of sternotomy?

### 1.1 The thymus and self-tolerance

#### 1.1.1 Functions of the thymus in self-tolerance

The thymic gland plays an essential role in T cell differentiation, establishing central tolerance. While both B and T cells recognize antigens specifically, they follow different pathways. T cells are incapable of recognizing antigens in their natural form and require maturation within antigen-presenting cells. There are two types of T cells: CD4 and CD8, each with distinct functions and development. CD4 cells assist in antibody formation and are involved in delayed-type hypersensitivity, recognizing antigens strictly in association with MHC class II. Conversely, CD8 cells play a role in viral infection defense, tumor rejection, and graft rejection, recognizing antigens in association with MHC class I.

The thymus is where the T cell receptor repertoire is generated, along with T lymphocyte maturation. During this differentiation process, a critical step is immune tolerance to self, which is relevant to MG.

Within the thymic cortex, the enabling environment for T-cell differentiation is created. As T cells migrate to the deep cortical areas, they express both CD4 and CD8. During their maturation, they develop into mature CD4+, CD4-, CD8+, and CD8- cells (8).

#### 1.1.2 The relationship between the thymus and MG

The role of the thymus in the pathogenesis of MG has been a subject of debate for many years and has undergone numerous studies. Structural changes in the thymus appear to be involved in the initial autoimmune response observed in MG. Autopsy analysis suggests that the thymus exhibits abnormalities in 80–90% of MG patients (9).

##### 1.1.2.1 Follicular lymphoid hyperplasia

Follicular lymphoid hyperplasia may be present in up to 70% of MG patients. It is frequently observed in women with early disease onset (age at onset less than 50 years with thymic hyperplasia). The architecture of the hyperplastic thymus remains intact. What stands out is the increased number of germinal centers in the medulla, similar to those observed in lymph nodes.

In follicular lymphoid hyperplasia, we can find B and T cells, plasma cells, and myoid cells. Myoid cells are the only cells capable of expressing antigenic determinants of the main immunogenic region of RACh (10). Thymocytes in culture can generate anti-RACh antibodies (AChR-Abs), supporting the disease’s pathogenesis (11).

##### 1.1.2.2 Normal thymus or thymic remnants

These are very common in cases of MG with late onset (age at onset greater than 50 years with thymic atrophy. Thymoma-associated MG. MG with anti-MuSK antibodies) and do not have a clear causal relationship or mechanism.

##### 1.1.2.3 Thymic tumor and thymoma

Approximately 10% of MG patients will present with a thymoma. They typically have AChR-Abs and exhibit more severe clinical manifestations but a similar prognosis to other MG patients (12).

In addition to MG, thymic tumors are associated with other autoimmune disorders. This can be explained by the fact that tumor cells present autoantigens, leading to the disruption of lymphocyte selection (13). A deficiency in AIRE gene expression and the selective loss of regulatory T cells have been described as the causes of negative selection and the upregulation of autoreactive T cells (14).

As previously mentioned, thymic tumors have been classified as malignant since the 2015 WHO classification (4). The treatment of thymic tumors should be individualized and approached from a multidisciplinary standpoint. Thymic tumor follow-up should be conducted for at least 10 years due to the tumor’s biology. This follow-up should run concurrently with that of MG, as MG exacerbations can sometimes result from tumor recurrence and should always be ruled out.

#### 1.1.3 Current evidence for surgery in MG

The ongoing controversy regarding the role of surgery led to a prospective, randomized, multicenter trial, the results of which were published in the NEJM journal in 2016 (7). The results were decidedly favorable in the group of patients who underwent surgery, showing improvements in symptoms, reduced reliance on corticosteroid therapy, and a lower number of recurrences at the three-year follow-up.

After the publication of these results, the role of the thoracic surgeon within the multidisciplinary treatment of MG regained significance. The findings from the NEJM publication, along with the new WHO classification, further emphasize the importance of thymectomy in the management of MG patients and those diagnosed with thymic tumors.

### 1.2 Types of surgical approach and recommended extent of thymectomy

#### 1.2.1 Types of surgical approach

*Transcervical approach*: As mentioned earlier, the inclusion of mediastinoscopy by Carlens (15) led to the resurgence of the transcervical approach in thymic gland resection. Zielinski et al. (16) have one of the largest published series of maximum thymectomies performed transcervically using the TEMLA (Transcervical Extended Mediastinal Lymphadenectomy) technique.

*Trans-sternal approach*: This category encompasses partial, total, or transverse sternotomy approaches. Through sternotomy, access is provided to both pleural cavities.

*Cervical and trans-sternal approach*: Combining both approaches allows us to explore the cervical area to rule out the presence of supernumerary thymic gland poles.

#### 1.2.2 Recommended extent of thymectomy

It’s essential to consider the anatomy of the thymic gland, being aware of its extrathoracic locations where supernumerary thymic gland poles can be found.

As mentioned previously, regardless of the approach chosen, a more extensive thymectomy yields better results, as described by Zielinski et al. (17).

Therefore, it is recommended to perform maximum thymectomy in both cases with associated thymic tumors and cases without them. A maximum extension is advised, avoiding thymomectomy, even in cases where there is no associated MG or other paraneoplastic syndromes, due to the non-negligible percentage of secondary tumor foci in other poles of the thymic gland.

#### 1.2.3 The emergence of thoracoscopic surgery in thymus surgery for MG

The introduction of thoracoscopic surgery in thymus surgery for MG marks a significant development. From a surgical perspective, one of the notable recent advances has been the gradual and successful integration of minimally invasive techniques in the field of thoracic surgery. Initially, VATS and subsequently RATS surgery have become the preferred techniques for most thoracic procedures.

#### 1.2.4 A critical issue in thymic surgery

One of the critical issues in thymic surgery is whether thoracoscopy can achieve the recommended resection and effectively remove most of the thymic gland.

Mantegazza (18) compared patients operated on for MG without associated thymic tumors, with 159 cases operated on using VATS versus 47 operated on with sternotomy. He focused on analyzing morbidity and mortality, observing a reduction compared to sternotomy. This study continued to address the concerns of critics of MG surgery, as the results of the 2016 study were not yet available.

Years later, in 2018, following the publication of the 2016 study in NEJM, Salim (19) presented a series of 50 patients, with 25 operated on using thoracoscopy and 25 using ministernotomy. The objective was to assess an even greater improvement in disease severity in MG in the thoracoscopy-operated group.

#### 1.2.5 The lack of comprehensive studies on thoracoscopic thymic gland surgical procedures

The scarcity of studies involving a substantial number of thoracoscopic thymic gland surgical procedures has prompted the initiation of the present project. The aim is to analyse our series of thymectomies conducted via thoracoscopy in comparison to those performed through sternotomy. This project seeks to contribute to the validation of the technique and to observe its impact on MG, specifically assessing how it affects symptomatology and the use of immunosuppressive treatments.

In this current study, we aim to analyse our series of thoracoscopic thymectomies and compare their outcomes with those of patients who underwent sternotomy. Additionally, we intend to assess the therapeutic outcomes on MG and thymic tumors in patients who underwent surgery, whether they had MG alone or in association with a thymic tumor.

### 1.3 Study hypotheses

- VATS thymectomy is a safe technique that results in reduced morbidity compared to sternotomy.
- VATS thymectomy is comparable to sternotomy in terms of improving the clinical condition and the need for corticosteroids in the medium and long term in patients with MG or other PNS.
- The presence of a concomitant thymic tumor in MG is a negative predictor for the MG’s progression.

## 2 Methodology

To validate our hypothesis, we designed an observational study conducted at a single-center referral center for MG. This study included 113 patients who had undergone thymectomy. Data were collected as follows: From 1990 to 2016, we retrospectively collected data from 40 patients who underwent sternotomy. From 2017 to 2021, we prospectively collected data from 73 patients who underwent VATS. In each group, we analyzed the following variables: surgical complications within 30 days, postoperative pain, and the duration of hospitalization. Additionally, for patients diagnosed with MG, we assessed the occurrence of myasthenic crises after surgery, the clinical response to thymectomy based on physical examination, and the impact on corticosteroid treatment at one, six, and twelve months following surgery.

### 2.1 Study population

#### 2.1.1 Selection of cases

##### 2.1.1.1 Retrospective cases

We identified cases from the databases of the Thoracic Surgery and Pulmonary Allergy departments at Santa Creu i Sant Pau Hospital, where thymectomy had been performed for the excision of mediastinal masses.

##### 2.1.1.2 Prospective cases

All patients diagnosed with MG who underwent VATS and those who underwent VATS or sternotomy with a diagnosis of mediastinal masses associated with MG or other paraneoplastic syndromes were prospectively included. Patients with a diagnosis of MG or other paraneoplastic syndromes not associated with thymic tumors were exclusively included in the prospective study.

Once the anatomopathological results were confirmed, and patients did not meet any exclusion criteria, they were included in the study.

### 2.2 Inclusion and exclusion criteria

#### 2.2.1 Inclusion criteria

- Patients diagnosed with a thymic tumor and associated paraneoplastic syndromes (MG or other) undergoing VATS thymectomy.
- Patients diagnosed with a thymic tumor and associated paraneoplastic syndromes (MG or other) undergoing sternotomy thymectomy.
- Patients diagnosed with MG or other paraneoplastic syndromes undergoing VATS thymectomy.
- Age between 16 and 85 years.
- Patients with thymic tumors without extrathoracic metastases.
- Patients who do not meet exclusion criteria.

#### 2.2.2 Exclusion criteria

- Patients with comorbidities that prevent them from undergoing surgical treatment.
- Patients with thymic tumors and extrathoracic metastases.
- Patients diagnosed with MG who do not meet the criteria for surgical indication.

The data search was conducted with a specific focus to achieve the established objectives.

In the primary cohort, the following data are recorded:

*Preoperative characteristics*: This includes information such as sex, age, the preoperative diagnosis that prompted the surgical intervention, and the surgical technique used for thymectomy. For patients diagnosed with MG or other paraneoplastic syndromes, the titration of autoantibodies in peripheral blood and whether or not corticosteroids were taken before surgery are recorded. If corticosteroids were taken, their doses in milligrams are also recorded.

*Postoperative characteristics*: These encompass the number of days of hospital stay and postoperative pain assessed using the VAS scale at discharge and one month after discharge.

*Surgical specimen*: This includes the definitive anatomopathological result. For patients with confirmed thymic tumors, the tumor stage according to the 8th TNM classification and the status of the neoplastic disease according to the last thoracic CT scan performed before the end of follow-up are also documented.

*Patients with a diagnosis of MG*: For these patients, data recorded include clinical worsening immediately after the intervention and the clinical assessment of the disease at the last recorded visit. For both variables, fatigue and muscle weakness were documented using the MG-ADL scale (20) described above in the study variables. The MG-ADL scale analyses the degree of difficulties that has the patient doing activities of daily living such as talking, chewing, swallowing, breathing, impairment of ability to brush teeth, or comb hair, impairment of ability to arise from a chair, double vision and eyelid droop. According to the results, the MG-ADL scale classifies the degree of MG from 0 to 3, being 0 a clinical situation with minimal symptomatology and being 3 severe symptomatology.

*Postoperative complications*: This section comprises details regarding mortality data, the reasons for patient exits during the entire follow-up period, the conversion rate in the VATS group, and complications associated with surgery within the initial 30 days post-surgery. Furthermore, a bivariate and multivariate analysis was conducted to identify independent predictors of complications following thymectomy, irrespective of the surgical approach used.

### 2.3 Analysis by subgroups

#### 2.3.1 Subgroup of patients according to the route of approach (VATS vs. sternotomy)

In this subgroup, the following characteristics were analyzed:

- *Preoperative characteristics*: Recording the median age in both the VATS group and the sternotomy group.
- *Postoperative characteristics*: Documentation of the number of days of hospital stay, levels of postoperative pain, and any postoperative complications.

#### 2.3.2 Subgroup of patients with a diagnosis of MG or other paraneoplastic syndromes (PNS)

Within this subgroup, the following characteristics are examined:

- *Preoperative characteristics*: Noting the surgical approach used and the titers of MG-specific autoantibodies in peripheral blood (AChR-Abs and anti-MuSK autoantibodies).
- *Postoperative characteristics*: Recording the duration of hospitalization, any changes in the dose of corticosteroid therapy if taken before surgery, any worsening of MG after surgery, and a clinical assessment at the last visit based on the MG-ADL scale.

#### 2.3.3 Subgroup of patients with a diagnosis of MG or other PNS

In this subgroup, the analysis includes the definitive anatomopathological diagnosis according to the pathology report and its relationship with clinical improvement according to the MG-ADL scale as a postoperative variable.

#### 2.3.4 Subgroup of patients with a diagnosis of thymic tumor

This subgroup includes the following:

- *Preoperative characteristics*: The surgical approach route used.
- *Postoperative characteristics*: The tumor stage and the status of the neoplastic disease at the last visit are documented.

The characteristics of the information source, the storage and database and the statistical analysis used are detailed below:

### 2.4 Sources of information

For this study, specific information in the variables section was collected from the medical records of the patients who consented to participate. This data was extracted from the SAP patient software, SAP Logon 7.40 CA ES x86N4, which has been in use at the Hospital de la Santa Creu i Sant Pau since 2011.

Patients who underwent surgery before 2011 had their medical records obtained in paper format from the hospital’s documentation department.

### 2.5 Data storage and processing

Throughout the study and during the targeted search for patient history information, the collected data were recorded on a designated form. These data were then entered into an Excel spreadsheet for storage and evaluation. Each patient was assigned an identification code.

The data were subsequently imported from the Excel spreadsheet and stored in the IBM SPSS program, version 22.0, for further analysis.

### 2.6 Expected sample size

No formal sample size calculation was conducted for this study. Since it is a retrospective and prospective descriptive study, all patients who underwent thoracic surgery and met the inclusion criteria during the estimated time frame were included. The last patient was included in December 2021 to allow for a two-month follow-up period for data analysis.

### 2.7 Statistical analysis

The statistical analysis was conducted by *Sail Biometria* – *Research and Logistics Consulting Services*^1^ and was funded by resources from the Thoracic Surgery Service at the *Hospital de la Santa Creu i Sant Pau* in Barcelona.

All study variables are summarized using descriptive statistics. For quantitative variables, the following statistics are presented: mean, standard deviation (SD), 95% confidence interval (95% CI) of the mean, median, range, and interquartile range. For qualitative variables, the summary includes absolute frequency (*n*) and relative frequency (%), categorized accordingly.

The distribution type of the variables was assessed, and their adherence to a Gaussian distribution was evaluated using the Kolmogorov–Smirnov test.

Homogeneity was examined among the following cohorts:

- Global sample (*N* = 113)
- VATS thymectomy vs. sternotomy
- 30-day post-surgery complications (No vs. Yes)
- Sample with MG or SPN
- Without thymic tumor (TmT) vs. with thymic tumor (TmT)
- With clinical improvement vs. without clinical improvement
- Preoperative corticosteroids vs. corticosteroids at 1-year follow-up

Statistical significance (value of *p*) was calculated using the appropriate statistical tests as follows:

- Fisher’s exact test for binary variables
- Chi-square test for variables with more than two categories
- Student’s *t*-test for continuous variables that follow a normal distribution
- Mann–Whitney test for continuous variables that do not follow a normal distribution
- Spearman’s correlation for ordinal variables

### 2.8 Ethical aspects

#### 2.8.1 Benefit–risk assessment of the research

Through this project, we hope to derive indirect benefits for patients who undergo this intervention:

- Awareness of the reduced morbidity associated with VATS thymectomy.
- Direct impact on MG or other paraneoplastic syndromes (PNS) in terms of clinical improvement and reduced immunosuppressant usage after surgery.
- Assessment of the therapeutic impact of VATS thymectomy at the oncologic level.

#### 2.8.2 Ethical, subject information, and informed consent considerations

The study was conducted in strict accordance with international ethical guidelines for medical research involving human subjects. The investigator ensured that the study adhered to the principles outlined in the Declaration of Helsinki.

Before beginning the study, the Ethics Committee of the *Hospital de la Santa Creu i Sant Pau* approved the study protocol, the information provided to the subjects, and the informed consent form used.

The investigator or a designated representative explained the study’s objectives, methods, and potential risks to the subject or their legal guardian or family member.

#### 2.8.3 Considerations on the treatment of biological samples

No biological samples were collected for this study.

#### 2.8.4 Data confidentiality

Regarding the confidentiality of study data, we adhered to the provisions outlined in Organic Law 15/1999 of December 13, 1999, concerning the “Protection of Personal Data.”

#### 2.8.5 Conflicts of interest

It is hereby declared that there are no conflicts of interest associated with the conduct of this research, whether by the principal investigator, directors, tutors, or other collaborators.

## 3 Results

### 3.1 Reference study population cohort

Between July 1990 and December 2021, a total of 113 thymectomies were performed at the Thoracic Surgery Service of the Hospital de la Santa Creu i Sant Pau. These surgeries were carried out either as treatment for mediastinal masses suspected of being tumors or as treatment for MG.

#### 3.1.1 Preoperative characteristics

##### 3.1.1.1 Sex and age

The majority of the patients who underwent surgery were women (*n* = 64; 56.6%). The median age of these patients was 53 years, with a range from 17 to 84 years.

##### 3.1.1.2 Preoperative diagnosis

**Figure 1:**
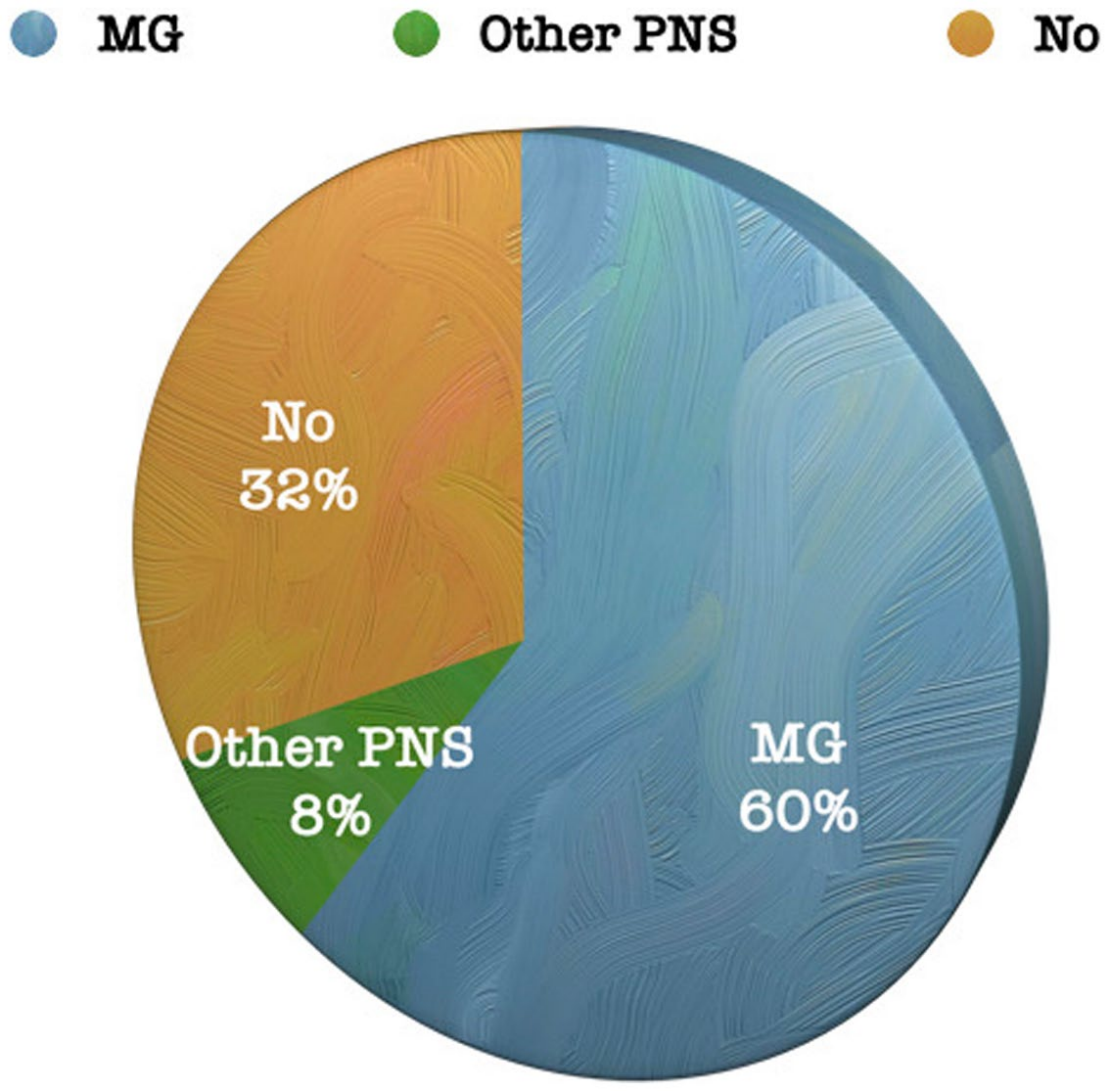
Percentage of patients diagnosed with PNS in the entire series.

Out of the 113 cases operated on (Figure 1):

- MG: clinically, 68 patients (60%) had a diagnosis of MG.
- Other paraneoplastic syndromes (PNS): Nine patients (8%) had PNS other than MG, including systemic lupus erythematosus (SLE), rheumatoid arthritis (RA), bullous pemphigoid, and polyneuropathy.

##### 3.1.1.3 Anatomical findings

From an anatomical perspective, CT imaging revealed some form of anatomical alteration in thymic cells in 100 patients (90%).

##### 3.1.1.4 Surgical technique

**Figure 2:**
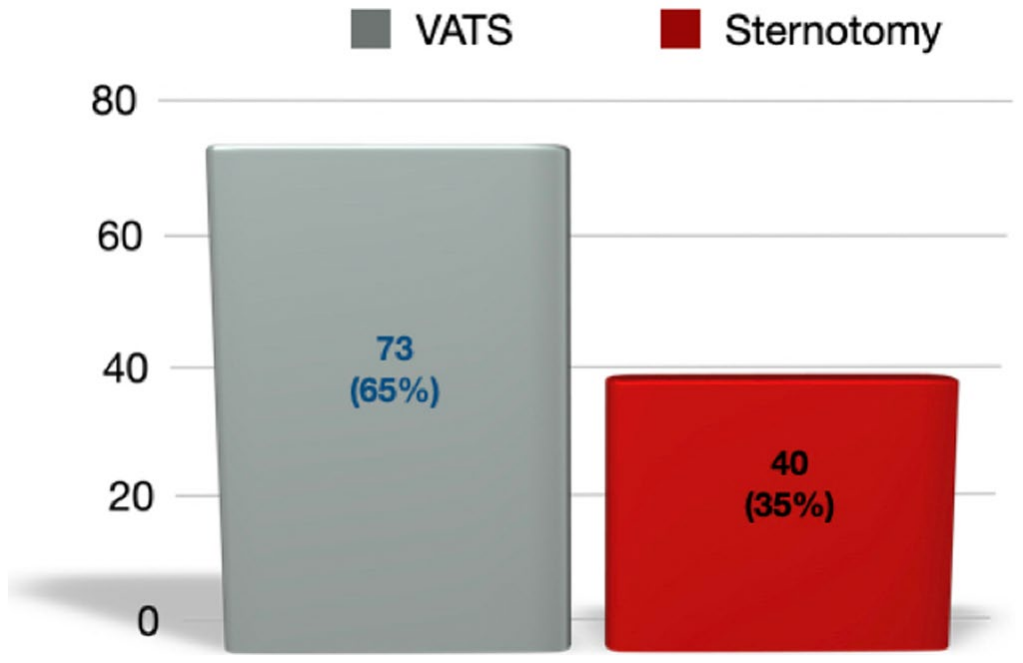
Total patients and percentage relative to the total cohort, depending on the surgical technique used.

The majority of patients underwent VATS (*n* = 73; 64.6%), with the right unilateral approach being the most commonly used (*n* = 52; 71.2%) (Figure 2).

##### 3.1.1.5 Corticosteroid treatment

Out of the group of patients with MG or other PNS, 47 patients (61%) were receiving corticosteroid treatment before surgery. Preoperative corticosteroid doses were recorded.

##### 3.1.1.6 Peripheral blood antibodies

In patients with MG or other PNS, peripheral blood was tested for AChR-Abs and anti-MuSK antibodies. These antibodies were found to be positive in 71 out of 77 patients (92%).

#### 3.1.2 Postoperative characteristics

##### 3.1.2.1 Anatomopathologic diagnosis

The anatomopathologic diagnosis after surgery yielded the following results:

- *Thymic hyperplasia*: A total of 23 patients (20.4%) underwent surgery with a diagnosis of thymic hyperplasia. All of these cases were patients who had a preoperative diagnosis of MG.
- *Primary thymic tumor*: Out of the total patients, 66 (58%) had a preoperative suspicion of a thymic tumor, and this diagnosis was confirmed after surgical resection. Among the patients with thymic tumors, 37% (*n* = 42) had MG or other associated PNS.
- *Other lesions*: In 11 cases (9.7%), the anatomopathological diagnosis after surgical resection confirmed a different diagnosis, including thymolipoma, thymic cysts, and ectopic parathyroid gland.

##### 3.1.2.2 Days of hospital stay

The median number of days of hospitalization after thymectomy in our series was 2 days, with a range from 1 to 40 days.

##### 3.1.2.3 Postoperative pain

Postoperative pain was assessed using the Visual Analog Scale (VAS). When analyzing the entire patient cohort, 43.8% of the patients classified their pain as mild at the time of discharge, and this percentage increased to 71.4% one month after the operation. Only 4.5% of the patients (*n* = 5) rated their pain as severe (VAS 8–10) one month after surgery.

##### 3.1.2.4 Treatment with corticosteroids after surgery

Regarding the reduction of corticosteroid treatment, approximately 50% of the patients who underwent surgery were able to progressively reduce their corticosteroid doses.

##### 3.1.2.5 Clinical assessment in specific consultation for MG or other associated NPS

Among the patients diagnosed with MG or other NPS, the majority experienced clinical improvement (*n* = 41; 53.2%) or clinical stability (*n* = 27; 35.1%) during follow-up consultations. Only 9 patients (11.7%) showed worsening of their condition after surgery.

#### 3.1.3 Postoperative complications

##### 3.1.3.1 Mortality

Out of the entire series of 113 patients, the mortality rate during follow-up was 9.7% (*n* = 11). Among these, one patient (9.1%) died in the immediate postoperative period (on the 3rd day).

##### 3.1.3.2 Conversion rate

The conversion rate was analyzed only in the VATS group. Among these patients, only 2 cases were converted to sternotomy, which corresponds to 2.7% of the VATS cases. In both cases, the cause of conversion was bleeding from a large vessel.

##### 3.1.3.3 Complications associated with surgery at 30 days post-surgery

The complication rate in the entire series was 24.8% (*n* = 28). The most frequent complication was phrenic nerve injury (*n* = 5; 17.9%), followed by hemothorax (*n* = 4; 14.3%) and pneumothorax (*n* = 4; 14.3%). There was only one empyema in the entire series associated with surgical intervention (3.6%).

Bivariate analysis identified two predictors of complications after thymectomy: the surgical approach and the presence of a thymic tumor.

##### 3.1.3.4 Presence of thymic tumor

Although the result was not statistically significant (*p* = 0.0751), there was a 21% increase in complications when the intervention was performed for the excision of a thymic tumor.

**Figure 3:**
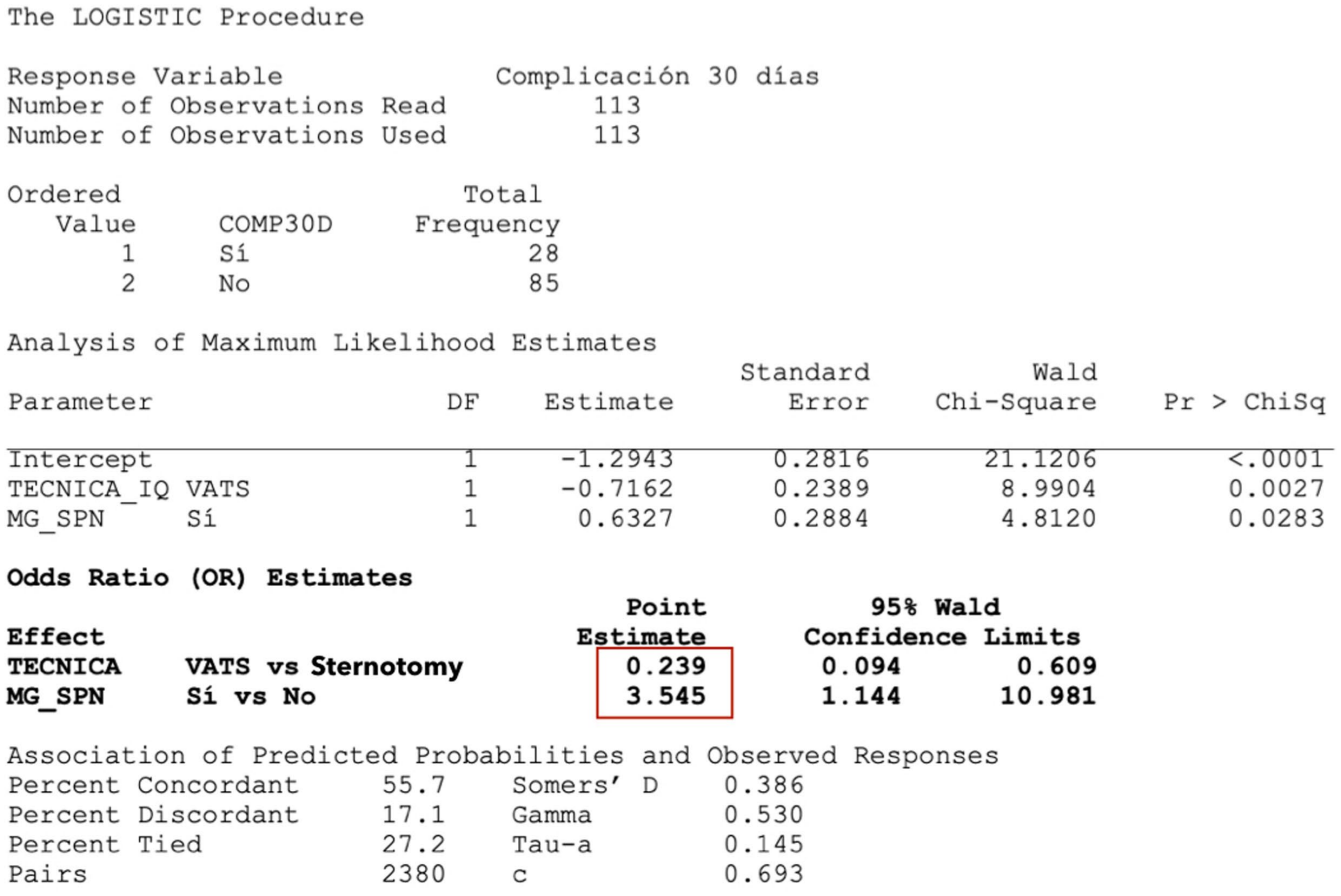
Predicators of post-surgery complications – multivariate logistic regression. In red, the higher probability of complications is marked in case of having MG or undergoing thymectomy by sternotomy.

After multivariate analysis, it was observed that the two factors acting as predictors of complications were the surgical approach and the presence of preoperative MG, whereas the presence or absence of a thymic tumor was not statistically significant (Figure 3).

1. *Surgical approach*: Patients undergoing VATS have a lower risk of complications (OR = 0.239; *p* = 0.0027) than patients undergoing sternotomy. In other words, patients operated by sternotomy are 4.2 times more likely (=1/0.239) to have more complications than patients operated by VATS.
2. *Presence of MG*: Patients with a preoperative diagnosis of MG have 3.5 times more risk of complications than patients without MG (*p* = 0.0283).

### 3.2 Patient cohorts according to the route of approach (VATS vs. sternotomy)

#### 3.2.1 Preoperative characteristics

##### 3.2.1.1 Age

The median age in both groups is similar (VATS = 52.6 [17.5; 84.1]; sternotomy = 54.1 [28.2; 81.8]), and this difference is not statistically significant.

#### 3.2.2 Postoperative characteristics

##### 3.2.2.1 Days of hospital stay

In the sternotomy thymectomy group, the median number of days of hospital stay was significantly higher (*p* < 0.0001) than in VATS patients (2.0 vs. 6.5, respectively).

##### 3.2.2.2 Postoperative pain

Patients who underwent VATS had less pain at discharge and one month after surgery, and this difference was statistically significant (*p* < 0.0001). Up to 62% of the VATS patients had mild pain at the time of discharge, whereas this percentage was only 7.7% in those who underwent sternotomy.

#### 3.2.3 Postoperative complications

##### 3.2.3.1 Complications associated with surgery at 30 days post-surgery

**Figure 4:**
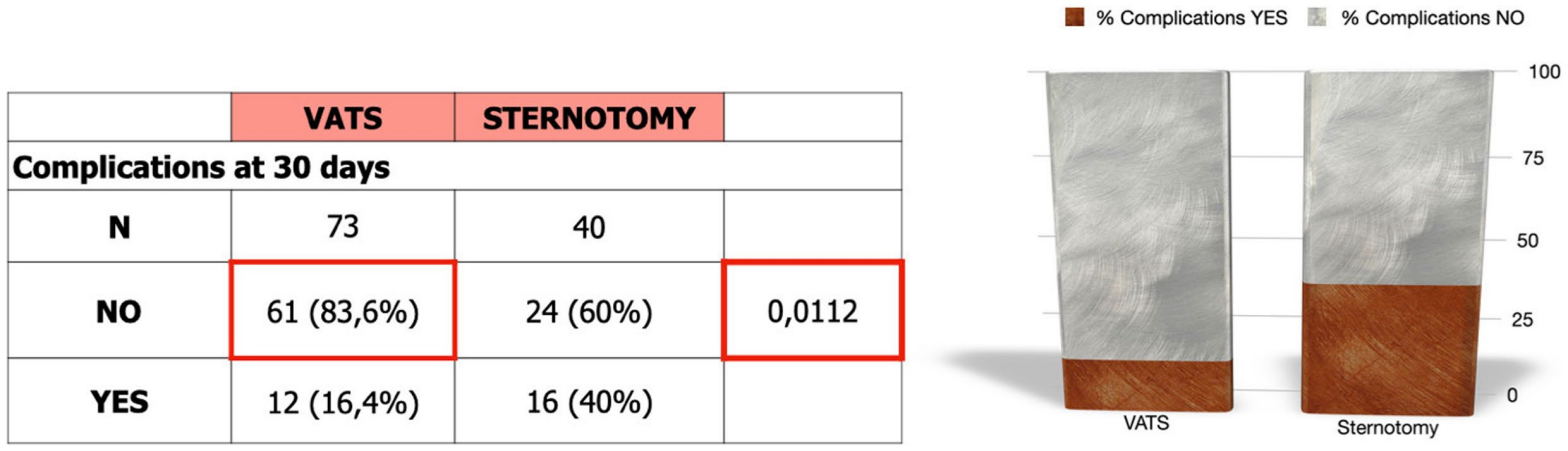
Percentage of complications depending on the surgical approach.

The rate of complications at 30 days post-surgery was significantly lower in patients operated by VATS (*p* < 0.0112). Among those who underwent sternotomy, 40% presented complications, with surgical wound infection (*n* = 8) being the most frequent. Forty percent of the patients in whom thymectomy was performed by sternotomy presented complications, the most frequent being surgical wound infection (*n* = 8). In the VATS group the most frequent complication was pneumothorax (*n* = 4) (Figure 4).

### 3.3 Cohort of patients with MG

The results of the cohort of patients with a preoperative diagnosis of MG are presented below. Of the total number of patients who underwent surgery (*n* = 113), 60% had a preoperative diagnosis of MG (*n* = 68; 60.1%).

The results of the variables that help us to assess the impact on the evolution of MG according to the type of approach by which thymectomy is performed are presented.

#### 3.3.1 Preoperative characteristics

- *Approach*: Among the patients diagnosed with MG (*n* = 68), 45 (66%) underwent VATS, and 23 (34%) underwent sternotomy.
- *Peripheral blood antibody title (AChR-Abs and anti-MuSK Abs)*: Most of the patients operated on with a diagnosis of MG had positive antibodies in peripheral blood regardless of the approach, with no statistically significant differences in both groups.

#### 3.3.2 Postoperative characteristics

- *Worsening of MG after surgery*: After surgery, there was a significantly greater increase in muscle weakness in patients who underwent sternotomy (48%) compared to those who underwent VATS (Table 1).
- *Myasthenic crisis*: There were no cases of myasthenic crisis in either group, regardless of the approach.
- *Clinical assessment in specific neuromuscular pathology consultation*: Patients who underwent VATS thymus excision showed a greater clinical improvement in their MG compared to those who underwent sternotomy, and this difference was statistically significant (*p* < 0.0044).
- *Change in corticosteroid dose preoperatively vs. one year after surgery*: We analyzed how many of the patients who were taking corticosteroids pre-surgery reduced their corticosteroid dose at one year. We observed that there was no statistically significant difference.
- *Days of hospital stay*: The data indicates that patients with an improvement in MG symptoms after the intervention had a shorter hospital stay (median 2.0 [1.0;13.0]).

**Table 1:** Worsening of MG after surgery according to the surgical approach.

	VATS	Sternotomy	*p*-value
Worsening of MG after surgery			
*N*	45	22	
No	45 (100%)	11 (50%)	<0.0001
Yes	0 (0%)	11 (50%)	

A multivariate analysis was attempted to determine which factors might be predictors of a better response after thymectomy in MG patients. However, this analysis was inconclusive due to the limitation of the sample size (*n* number).

### 3.4 Cohort of patients with MG or other syndrome associated with THYMIC lesion

The following are the results of the cohort of patients with a preoperative diagnosis of MG or other SPN, who also present a thymic lesion confirmed after anatomopathological study.

The results of the variables that help us to assess the impact on the evolution of MG according to the thymic lesion present are presented.

#### 3.4.1 Postoperative characteristics

In the cohort of 77 patients with MG or other PNS, 23 cases showed The presence of thymic hyperplasia, 42 cases showed The presence of a thymic tumor, and The remaining 12 cases showed other thymic lesions or a normal thymus (Table 2).

- *Diagnosis of thymic hyperplasia*: Patients whose thymectomy confirmed the diagnosis of thymic hyperplasia had a better clinical evolution of MG.
- *Thymic tumor*: Among the patients who underwent surgery and had a thymic tumor confirmed after thymectomy, the clinical evolution of MG was worse.

**Table 2:** Relationship between the presence of hyperplasia and the clinical evolution of MG.

MG or other PNS	Improvement: No	Improvement: Yes	*p*-value
Pathological results			
*N*	37	40	
No hyperplasia	3 (8%)	9 (22.5%)	0.0050
Hyperplasia	7 (19%)	16 (40%)	
Thymic tumor	27 (73%)	15 (37.5%)	

### 3.5 Population cohort of patients diagnosed with THYMIC tumor

The results of the cohort of patients diagnosed with thymic tumors, confirmed by anatomopathologic results, are presented below. Among the analyzed cohort of patients (*n* = 103), 66 patients were diagnosed with thymic tumors.

#### 3.5.1 Preoperative characteristics

##### 3.5.1.1 Approach

Of the 66 patients diagnosed with thymic tumor, 26 (39%) were operated by VATS and 40 (61%) by sternotomy.

#### 3.5.2 Postoperative characteristics

##### 3.5.2.1 Thymic tumor staging

We observed that most of the tumors presented a pT1N0 stage, both in the group operated by VATS (*n* = 29; 63%) and those operated by sternotomy (*n* = 11; 52.4%), with no statistically significant differences.

##### 3.5.2.2 Neoplastic disease status

There were statistically significant differences (*p* < 0.0001) in terms of local or distant recurrence depending on the approach. In patients operated by VATS with a diagnosis of thymic tumor (*n* = 26), there was no evidence of local or distant recurrence. In the cases operated by sternotomy (*n* = 39), 46.2% presented local or distant recurrence.

## 4 Discussion

Thymectomy is one of the most common surgical procedures in thoracic surgery departments and has been widely used for various diseases. It is likely one of the earliest procedures in thoracic surgery to adopt minimally invasive techniques. After the initial positive publications, the trend of minimally invasive surgery in thoracic procedures, including thymus surgery, has continued to grow.

However, despite the early favorable reports, some researchers have raised questions about the quality of VATS thymectomy. They have pointed to limitations in the size and heterogeneity of published series, leading to ongoing debates in the field.

These debates primarily revolve around three key issues:

- The validity of VATS thymectomy vs. sternotomy: The effectiveness and safety of VATS thymectomy compared to the traditional sternotomy approach have been a subject of contention.
- The controversy over thymectomy for MG without thymic tumors: The role of thymectomy as a treatment for MG, especially in patients without thymic tumors, remains a topic of debate.
- The oncologic radicality achieved with VATS thymectomy: Concerns have been raised about whether VATS thymectomy can provide the same level of oncologic completeness as sternotomy, particularly for thymic tumors.

In light of these ongoing debates, our study was designed to investigate these issues by examining our patient cohort and providing insights into the current state of thoracic surgery.

### 4.1 Representativeness

Our study cohort includes a group of patients who underwent thymectomy at our medical center. While it is a study conducted in a single medical facility, it is important to note that our center specializes in the treatment of MG. This specialization means that we receive a higher number of patients requiring thymectomy evaluation compared to other centers. However, due to the relatively low prevalence of both MG and thymic tumors, the total number of patients included in our study, collected over a span of 30 years, is only 113 cases. This highlights the challenges in recruiting a larger sample size. Nevertheless, it’s worth noting that the sample size in our study is comparable to or even larger than other published reference series (19).

Another crucial aspect to consider within our series is that the cases treated with sternotomy belong to the earlier series. Consequently, the outcomes observed should not be solely attributed to the quality of the surgical technique but should also take into account the advancements in perioperative care processes that patients have undergone over time.

### 4.2 Validity of VATS thymectomy compared to sternotomy

In the early 2000s, numerous publications highlighted the advantages of VATS over sternotomy for thymus resection (21, 22). Due to the relatively small number of patients included in each of these series, several meta-analyses were conducted to assess the quantity and quality of the evidence available.

In the first meta-analysis by Yang et al. published in 2015, they analyzed 14 publications out of more than 200, which met the necessary quality criteria for drawing conclusions regarding the comparison of both techniques. These 14 publications encompassed a total of 1,087 patients, with 587 undergoing VATS and 400 undergoing sternotomy. Their results demonstrated that VATS led to a reduction in hospital stay, decreased intraoperative blood loss, and a lower rate of complications, all of which were statistically significant (23).

A year later, in 2016 (24), a new meta-analysis was conducted. This analysis examined 12 articles selected from a pool of 162 (with only two being prospective studies) that compared both techniques. In this meta-analysis, it is again apparent that patient recruitment is limited, with the longest series being published by Julissa et al. in 2013 (25). They conclude that in those who underwent VATS there was a reduction in intraoperative blood loss, a shorter hospital stay and an overall reduction in complications. On the other hand, no statistically significant differences were found in the rate of postoperative infections.

Although the advantages of VATS thymectomy over sternotomy seem apparent, a study by Orsini et al. (26) in 2015, involving 278 patients from the EPITHOR database in France, presented a different perspective. Among these patients, 161 underwent sternotomy, and 116 underwent VATS thymectomy. This study stands out for its relatively large sample size but reaches conclusions that emphasize the challenges of making definitive claims about the superiority of one technique over the other due to the inherent heterogeneity between the two groups. Despite the growing adoption of VATS, this study could not conclusively demonstrate its clear superiority.

Upon analyzing our own series of patients, several postoperative variables clearly favored VATS thymectomy:

- *Hospital stay:* VATS patients experienced a remarkable reduction in hospital stay, with a median of only 2 days. This figure is notably lower than in other VATS series, where the median stay, although shorter than sternotomy, typically averaged around 4 days (26).
- *Postoperative pain*: VATS was associated with reduced postoperative pain, both immediately after surgery and one month after discharge. A significant finding was the decreased need for opioid pain relief in the first 24 h post-surgery.
- *Complications at 30 days post-surgery*: The rate of complications was significantly lower in the VATS group. It’s important to note that the existing literature does not consistently report a reduction in complications, which can be attributed to the heterogeneity among patient groups in different studies (23, 26, 27).

In an effort to identify potential predictors of complications, a multivariate logistic regression model was applied. This analysis revealed that, apart from the surgical approach where VATS clearly had an advantage, the presence of preoperative MG, regardless of the approach, acted as a predictor of complications. This association is logical, as many of these patients are under pharmacological immunosuppression, making them more susceptible to post-surgery infections. In our series, contrary to the findings in the meta-analysis by Kang Qi et al. (24), the infection rate was significantly higher in the sternotomy group. This was attributed not only to the higher prevalence of MG in this group (58%) but also to the increased infection risk associated with sternotomy.

To eliminate potential selection biases favoring these results, we examined whether there were significant age differences between both groups. It was found that both groups had a similar median age.

It’s important to emphasize that the outcomes related to the variables mentioned above are not solely determined by the surgical technique itself. In the current medical landscape, there is a growing trend toward the integration of prehabilitation programs. These programs aim to optimize various preoperative factors, leading to a reduction in complications, improved pain management, and earlier discharge. Such programs are increasingly becoming a standard practice in the field of surgery, contributing significantly to the overall patient care and recovery process.

### 4.3 Thymectomy in MG

#### 4.3.1 Indication

The use of thymectomy in the treatment of MG has been a subject of ongoing debate, particularly when there is no associated tumor. In 2000, Grohnset et al. (28) conducted an analysis of 28 non-randomized studies comparing outcomes between thymectomized and non-thymectomized MG patients. While the study concluded that thymectomy had a beneficial effect on clinical improvement in MG patients, it faced criticism due to the non-randomized nature of the trials and the heterogeneity of the groups being compared.

This controversy was reflected in the 2010 guidelines for the treatment of neuromuscular disorders, which excluded thymectomy from therapeutic recommendations due to inconsistent selection criteria and variability in response assessment criteria following the procedure.

Despite the trend toward discontinuing thymectomy in MG, Kauffman et al. (29) published the results of a retrospective series of over 1,000 MG cases in 2016, demonstrating a 47% improvement in symptoms following thymectomy.

To address ongoing uncertainties, the first prospective, randomized, multicenter trial, known as the MGTX Trial, was designed to compare two groups: one group underwent transsternal thymectomy in addition to prednisone treatment, while the second group received prednisone treatment alone. The results favored the surgical group.

The analysis of the results from our series, which included 77 patients diagnosed with MG who underwent surgery, aligns with the findings of the MGTX Trial. It appears that patients who underwent thymectomy experienced positive outcomes in terms of MG symptom improvement and remission, consistent with the trial’s conclusions. Showed an improvement in MG symptoms regardless of the approach used.

On the other hand, we could not demonstrate a significant reduction in corticosteroid intake. Probably the limited number of patients treated with corticosteroids and the short follow-up time have influenced the outcome with respect to corticosteroid therapy.

#### 4.3.2 Impact according to the approach route

Once the importance of thymectomy in treatment was reestablished, a new debate emerged regarding the surgical approach. In the MGTX Trial, all patients underwent sternotomy, leaving uncertainty about whether VATS could achieve comparable results. Concerns were based on findings from a study by Kaufman et al., which noted that only patients who underwent sternotomy showed clinical improvement.

Studies like the one published by Evoli et al. (30) indicated that, in addition to the improvements in morbidity associated with the technique, the impact of VATS thymectomy in MG was similar to results obtained with sternotomy.

In contrast, a recent study by Raja et al. (31) concluded that more information is needed to confirm that the reduced morbidity associated with VATS translates into an improvement in MG symptoms. Beyond debates about the surgical approach, Jaretzki and Sonett suggested that the crucial factor lies not in the approach itself but in the extent of resection, recommending excision of the cervical poles of the thymus to avoid leaving up to 25% of the gland behind.

Based on our results, it appears that thymectomy has a more significant impact on MG symptoms in patients undergoing VATS. Clinical assessments showed greater symptom improvement in this group. However, it’s important to acknowledge potential selection bias when interpreting these results. All patients in our series who underwent sternotomy with a diagnosis of MG also had a concurrent diagnosis of another autoimmune disease, potentially influencing the outcome. Thymic tumor. When analyzing the predictors of response we will see that the presence of thymic tumor acts as an unfavorable predictor.

On the other hand, there was no significant difference in the reduction of corticosteroids one year after the intervention.

#### 4.3.3 Predictors of MG response to thymectomy

Several predictors of a positive response to thymectomy in patients with MG have been identified. Reviewing the literature, factors associated with a better response include:

- Younger age at the time of surgery.
- Shorter duration of MG symptoms before thymectomy.
- Presence of thymic hyperplasia or thymoma as opposed to normal thymus.
- Positive AChR-Abs status.
- Lack of severe bulbar symptoms.
- Lower prednisone dose at the time of surgery.
- Preoperative Osserman stage I or II.
- Female gender.

In our series, we have identified a selection bias that prevents us from determining whether the presence of certain factors acts as predictors of a positive response or not. For instance, regarding the levels of ACs (antibodies) in peripheral blood, over 90% of our patients had positive values before surgery. This was because it was already a selection criterion for surgery. Most of the patients, whether they had MG associated with a thymic tumor or not, tested positive for these antibodies. Only two patients underwent surgery with negative ACs, and their outcomes were favorable. However, this result was not statistically significant due to the small sample size of two cases.

In terms of the age of the cohort of MG patients without associated thymic tumors, their median age was 42 years. Here, too, there is a selection bias since this criterion was considered before referral to our office.

We have been able to confirm that the presence of thymic hyperplasia, as confirmed in the definitive anatomopathological study, is a favorable factor for a positive response. This finding is statistically significant.

The presence of a thymic tumor, according to existing literature, tends to be an unfavorable factor in MG patients. This concomitance often occurs at an older age and is associated with a worse prognosis in terms of MG progression (32).

Our analysis of 42 patients who had both MG and thymoma confirms that the presence of a thymic tumor acts as an unfavorable factor. These patients tend to have a more challenging clinical course, requiring a progressive increase in immunosuppressive medication to manage their symptoms. Furthermore, when you examined the relationship between thymic tumor size and patient outcomes, you found that patients with larger pTNM tumors had a worse prognosis.

It’s worth noting the association between tumor recurrence and the worsening of MG. You observed that the timing of disease recurrence coincided with MG deterioration or an increased need for corticosteroids. Therefore, in patients with thymoma who experience MG worsening, it’s crucial to investigate the possibility of tumor recurrence through imaging. In some cases, this recurrence may manifest in extrathoracic locations.

### 4.4 Oncologic outcomes by route of approach

From the oncological point of view, the use of new minimally invasive techniques such as VATS should not reduce the guarantees of the oncological quality of the resection (33). The application of new techniques should follow the same oncological principles and also, if possible, offer a reduction in the associated morbidity.

Different authors support the fact that there may be a higher rate of recurrence in cases operated by VATS for two main reasons (34):

- Excessive manipulation of the tumor due to the reduction of the operative field, which can cause capsular rupture, especially in large tumors.
- The excision of the specimen through small incisions must be careful since it can produce compression of the tumor, capsular rupture, and as a consequence tumor dissemination.

Our results do not agree with these critical statements regarding VATS. We observed that during follow-up, more than 40% of patients who underwent sternotomy had local or distant recurrence, whereas in patients who underwent VATS the rate was 0%.

The first reason we can associate with the difference in oncologic outcomes is that the tumors operated on by VATS corresponded to smaller tumors. However, upon analyzing the results, we observed that there were no differences in terms of pathologic TNM staging in both groups.

Multiple factors may explain this difference, but we want to highlight the following:

1. After the update in the WHO classification published in 2015, all thymic tumors are now considered malignant tumors. This change led to a shift in our overall approach to the diagnosis and treatment of these tumors, particularly regarding surgical treatment. In the past, the erroneous classification of these tumors as benign meant that more extensive surgery was not always considered. Instead, suboptimal thymus resections were often offered, which may not have been oncologically ideal point of view.

Currently, we understand that one of the primary prognostic factors for thymic tumors is achieving a complete resection during surgery. En bloc resections, involving the removal of the tumor along with potentially infiltrated structures, are considered the minimum acceptable approach to avoid suboptimal treatment. Due to the low prevalence of these tumors and the evolving approach to their diagnosis and treatment, the idea of creating homogeneous, multicenter, international databases has gained momentum. In 2010, the ITMIG group proposed such a database with the goal of assisting in the reorientation of diagnostic and therapeutic protocols for thymic tumors. The ITMIG database is now recognized as the most extensive thymic tumor-specific database globally, with more than 10,800 registered cases. Until relatively recently, the assessment of thymic tumor treatment did not involve multidisciplinary committees. Instead, it was divided into surgical treatment for potentially resectable cases and oncologic treatment for unresectable cases. However, our current understanding emphasizes the importance of precise diagnosis and preoperative staging. We must also recognize that, in some cases, chemotherapy and radiotherapy will play a crucial role in treatment, in addition to surgery.

This underscores the necessity for specialized reference units equipped with specific multidisciplinary committees. These committees should be led by professionals experienced in the diagnosis and treatment of thymic tumors, as emphasized in the guidelines published by the National Comprehensive Cancer Network (NCCN) (35).

1. Despite being classified as malignant tumors, thymic tumors exhibit slow growth rates due to their unique biology. Consequently, it is recommended to extend the follow-up period to 10 years to minimize the chances of local or distant recurrence. Considering this factor, it’s important to note that our prospective series has not yet reached the recommended minimum follow-up period. The first VATS thymectomy case in our series was performed in 2017. Therefore, further research will be necessary to validate the observed recurrence rates in our VATS series. Recognizing the limitations of the population studied, the results obtained from our study lead us to conclude that, based on our experience, VATS thymectomy is a safe technique associated with a reduced incidence of postoperative complications compared to sternotomy. In our working group, we can confidently state that performing thymectomy using minimally invasive techniques is both safe and effective.

## 5 Conclusion

- VATS thymectomy demonstrates a lower rate of intraoperative and postoperative complications, reduced morbidity, and a shorter postoperative length of stay when compared to sternotomy thymectomy.
- VATS thymectomy leads to a favorable impact on MG symptoms.
- Patients with MG who undergo sternotomy experience greater postoperative muscle weakness than those undergoing VATS.
- Patients with MG undergoing VATS achieve better long-term control of myasthenic symptoms than those undergoing sternotomy.
- These results underscore the importance of continuing thymectomy using minimally invasive techniques due to their superiority in our series. Additionally, they emphasize the need for enhancements in perioperative care programs.
- The presence of thymic hyperplasia in the definitive pathology results has been identified as a favorable predictor of clinical improvement. Therefore, diagnosing thymic hyperplasia via CT should serve as a selection criterion when considering thymectomy for MG patients.
- Conversely, the presence of thymic tumors negatively affects the clinical course of MG.
- Patients undergoing VATS have lower rates of local and distant recurrence compared to those undergoing sternotomy.

### 5.1 Limitations of the study

- The study has limitations due to the low prevalence of both MG and primary thymic gland tumors.
- The non-homogeneity of the series is another limitation, attributed to the incorporation of new surgical approaches, changes in the global approach to treating MG and thymic tumors, and improvements in perioperative care in recent years. Each of these factors can independently influence the results obtained.
- Interpretation of oncologic results for VATS thymectomy should be approached cautiously, as these tumors require long-term follow-up due to their distinct biology.

### 5.2 Future prospects

We must increase the number of randomized trials with homogeneous groups of patients that will allow us to improve the knowledge of both pathologies within the medical society. This will lead to better diagnostic and therapeutic management at an early stage of the disease. To achieve this objective, it is necessary to create reference, multicenter and international groups that allow the creation of databases of a greater number of patients.

The lines of work should be focused on research in both the experimental and clinical (diagnostic and therapeutic) fields. The choice of surgical approach is simply a small grain of sand on a vast beach of ignorance.

## Ethics statement

The studies involving humans were approved by Ethics Committee of Hospital de la Santa Creu i Sant Pau. The studies were conducted in accordance with the local legislation and institutional requirements. The participants provided their written informed consent to participate in this study. Written informed consent was obtained from the individual(s) for the publication of any potentially identifiable images or data included in this article.

## Author contributions

JT: Data curation, Investigation, Methodology, Supervision, Validation, Visualization, Writing – original draft, Writing – review & editing. EM: Data curation, Validation, Visualization, Writing – review & editing. JB: Formal analysis, Investigation, Methodology, Supervision, Writing – review & editing. GP: Data curation, Visualization, Writing – review & editing. AL: Writing – review & editing, Data curation. MG: Data curation, Writing – review & editing. JH: Data curation, Writing – review & editing. AM: Formal analysis, Methodology, Supervision, Visualization, Writing – review & editing.

## Footnotes

^1^ https://www.sail-biometria.com
